# Case report: A *Saprochaete clavata* (*Magnusiomyces clavatus*) severe infection effectively treated with granulocyte transfusion in a young patient with myeloid sarcoma

**DOI:** 10.3389/fonc.2022.970188

**Published:** 2022-09-15

**Authors:** Gianmario Pasqualone, Elisa Buzzatti, Raffaele Palmieri, Arianna Savi, Maria Rosaria Pascale, Beatrice Borsellino, Luca Guarnera, Francesco Buccisano, Maria Teresa Voso, Luca Maurillo, Giuseppe Sconocchia, Adriano Venditti, Maria Ilaria Del Principe

**Affiliations:** ^1^Hematology, Department of Biomedicine and Prevention, Università degli studi di Roma Tor Vergata, Rome, Italy; ^2^Institute of Translational Pharmacology, Department of Biomedical Sciences, National Research Council (CNR), Rome, Italy

**Keywords:** acute myeloid leukemia, invasive fungal infection, granulocyte transfusion, *Saprochaeta clavata*, *Magnusiomyces clavatus*, case report, myeloid sarcoma (MS)

## Abstract

Myeloid sarcoma is a hematologic malignancy consisting of extramedullary tissue involvement by myeloid blasts, usually considered as acute myeloid leukemia and treated accordingly. The disease itself, together with chemotherapy and disease-associated factors, may have an impact in increasing the risk of developing severe and frequently life-threatening infections. Herein, we describe the case of a patient with a right breast skin lesion, histologically diagnosed myeloid sarcoma, who developed a severe disseminated fungal infection by *Saprochaete clavata* (*Magnusiomyces clavatus*), during the first consolidation course of chemotherapy. Despite maximum antifungal therapy, the infection progressed and the fungus continued to be isolated until granulocyte transfusion therapy was initiated. Our experience suggests that patients with profound and long-lasting neutropenia could benefit from granulocyte transfusions as additional therapy in severe fungal infections resistant to broad-spectrum antimicrobial therapy.

## Introduction

Acute myeloid leukemia (AML) is a malignant disorder caused by clonal expansion of hematopoietic cells abnormally or poorly differentiated, called blasts. These cells infiltrate and accumulate preferentially in the bone marrow (BM), with consequent impairment of normal hemopoiesis (1). AML categorization and classification rely on morphologic, immunophenotypic, cytogenetic, and molecular genetic analyses (2, 3). AML could be preceded or concurrent with an extramedullary localization of blasts, a condition defined as myeloid sarcoma (MS) (4).

MS is a rare malignant disease that could occur virtually in any anatomical site, most frequently in connective/soft tissues, gastrointestinal (GI) tract, spleen, and lymph nodes. Sanctuary sites, such as the central nervous system (CNS) and testis, could also be affected (4–6). Diagnosis can be challenging, and misdiagnosis is frequently reported: imaging (mostly computed tomography [CT], magnetic resonance imaging, F^18^-fluorodeoxyglucose-positron-emission tomography [FDG-PET]) and most importantly immunohistochemical/cytochemical analysis are needed to confirm the clinical suspicion (5, 7). Therapeutic strategies in MS should consider tumor size and site, performance status (PS), patient’s age, and comorbidities. To date, systemic chemotherapy (CHT) is considered the backbone of treatment.

Although data regarding the prognosis of MS in the setting of AML are limited and somewhat conflicting, it seems that the presence of extramedullary localization does not represent an independent prognostic feature of the underlying AML, so the outcome depends on the prognostic stratification of AML itself (4, 8, 9).

Patients diagnosed with hematological malignancies are subjects vulnerable to common and opportunistic infections because of several risk factors: besides the underlying malignancy, therapy-related immunosuppression, neutropenia, presence of central venous catheter (CVC), and the use of broad-spectrum antimicrobial therapy are additional elements to be considered. In such a context, invasive fungal infections (IFIs) represent a serious and life-threatening complication, requiring a timely identification and treatment.

*Saprochaete clavata*, formerly *Geotrichum clavatum* (often misidentified for *Saprochaete capitata*, formerly *Geotrichum capitatum/Magnusiomyces capitatus*, because of their close similarity), has recently emerged as a new threat among hemato-oncological patients with reported mortality rates up to 80%, with AML identified as the most common underlying malignancy (10–12). *S. clavata* infection is almost invariably identified in advanced phases of dissemination (positive blood cultures or organ infiltration). Despite that the site through which this pathogen enters the bloodstream is frequently unclear, respiratory or GI systems are likely to be preferential sites of colonization. Consequently, CHT-induced damage of the mucosal barrier promotes pathogen translocation (10, 12). There is no standard treatment for *S. clavata*, and no standardized European Committee on Antimicrobial Susceptibility Testing antifungal breakpoints are determined for this pathogen: therapy should be based on expert opinion and accurate interpretation of susceptibility tests. Antifungal combination therapy could represent an effective choice, but hematological recovery appears critical to enhance and accelerate clinical resolution (12, 13).

In this regard, cases of prolonged neutropenia and infection by multidrug-resistant or hard-to-treat pathogens might benefit from granulocyte transfusion (GTX). GTX is not a new therapeutic approach, but the interest around this strategy has consistently diminished due to the availability of new antibiotic and antifungal therapies (13, 14). However, several reports and case series have pointed out the effectiveness of this procedure, especially in situations of delayed neutrophil count recovery and failure of antimicrobial therapies (15, 16).

Here, we describe the case of a patient diagnosed with MS, who underwent systemic CHT and developed a severe IFI during the post-consolidation neutropenic phase. Despite maximal antifungal therapy, *S. clavata* continued to be isolated from blood cultures with progressive worsening of the patient’s conditions. The introduction of GTX promptly reverted the clinical picture, with resolution of the infection.

## Case description

The patient is a 24-year-old woman, who was referred to primary care in November 2020 because of a painless, indurated, purplish skin plaque of the right-side breast. Past medical history was uneventful, and she was only on oral contraceptives.

In May 2021, a punch biopsy of the lesion was diagnostic for MS, with immunohistochemistry positive for CD117, cKit, CD4, CD56, CD33, PGM1, and Bcl2 and negative for CD34, MPO, CD20, CD3, CD23, Tdt, and CD79a.

In June 2021, she was referred to our Institution and underwent an FDG PET-CT scan showing a slight and unspecific bilateral and symmetric nasopharyngeal uptake in addition to the known skin lesion. Thus, a biopsy of the nasopharynx was collected for diagnostic purposes. No fatigue, fever, night sweats, weight loss, or other constitutional symptoms were reported. Complete blood count (CBC) showed unremarkable results, and BM aspiration (BMA) showed a negative result for leukemic involvement, with a normal karyotype identified upon cytogenetic analysis. Lactate dehydrogenase and hepatic, renal, and coagulation profiles showed normal results. To relieve emerging pain and swelling, local radiotherapy of the breast skin lesion was started (24 Gy over 12 fractions).

In July 2021, histological examination of the nasopharyngeal biopsy confirmed the MS localization expressing the same immunohistochemical profile of the primary breast lesion. In August 2021, the patient was hospitalized to proceed with restaging and to undergo systemic CHT.

A BM reassessment showed a 60% blast infiltrate with a CD45+, CD34+, CD33+, CD117+, CD64+, anti-HLA-DR +, CD4+, and CD56+ immunophenotypic profile. The cytogenetic study revealed the appearance of chromosome 4 trisomy (47, XX,+4 in 10/15 analyzed metaphases). The analysis of common fusion genes and mutations (BCR/ABL, RUNX1/RUNXT1, DEK/NUP214, CBFβ/MYH11, FLT3-ITD/TKD, and NPM1) showed a negative result for molecular abnormalities. The morphologic and phenotypic examination of cerebral spinal fluid excluded CNS involvement. According to the 2017 European LeukemiaNet recommendations, intermediate-risk AML with concurrent MS was the final diagnosis (2, 4).

Consequently, an FLAI induction CHT regimen (5 days of fludarabine 30 mg/m^2^ daily; 5 days of cytarabine 2,000 mg/m^2^ daily; 3 days of idarubicin 10 mg/m^2^ daily) was initiated. Prophylactic posaconazole and filgrastim were given until neutrophil recovery.

During hospitalization (27 days), a single episode of febrile neutropenia occurred, which was effectively treated with a broad-spectrum antibiotic therapy (piperacillin/tazobactam 4.5 g/8 h for 14 days, with no isolates on blood cultures). Oral grade III mucositis (17) also occurred and resolved before discharge.

Upon full CBC recovery, the patient was reassessed and found to be in a BM complete remission (CR), with a complete clearance of the breast skin lesion.

Accordingly, she received a second course of FLAI as a consolidation therapy. Filgrastim (5 µg/kg/day) was started on day 9 from the first day of FLAI. On day 15, an episode of febrile neutropenia was recorded and treated with a broad-spectrum antibiotic therapy (piperacillin/tazobactam 4.5 g/8 h for 4 days, then meropenem 1 g/8 h because of persistence of fever). Fever was associated with elevation of procalcitonin (PCT: 2.38 ng/ml, reference values: 0.01–0.50 ng/ml).

**Figure 1:**
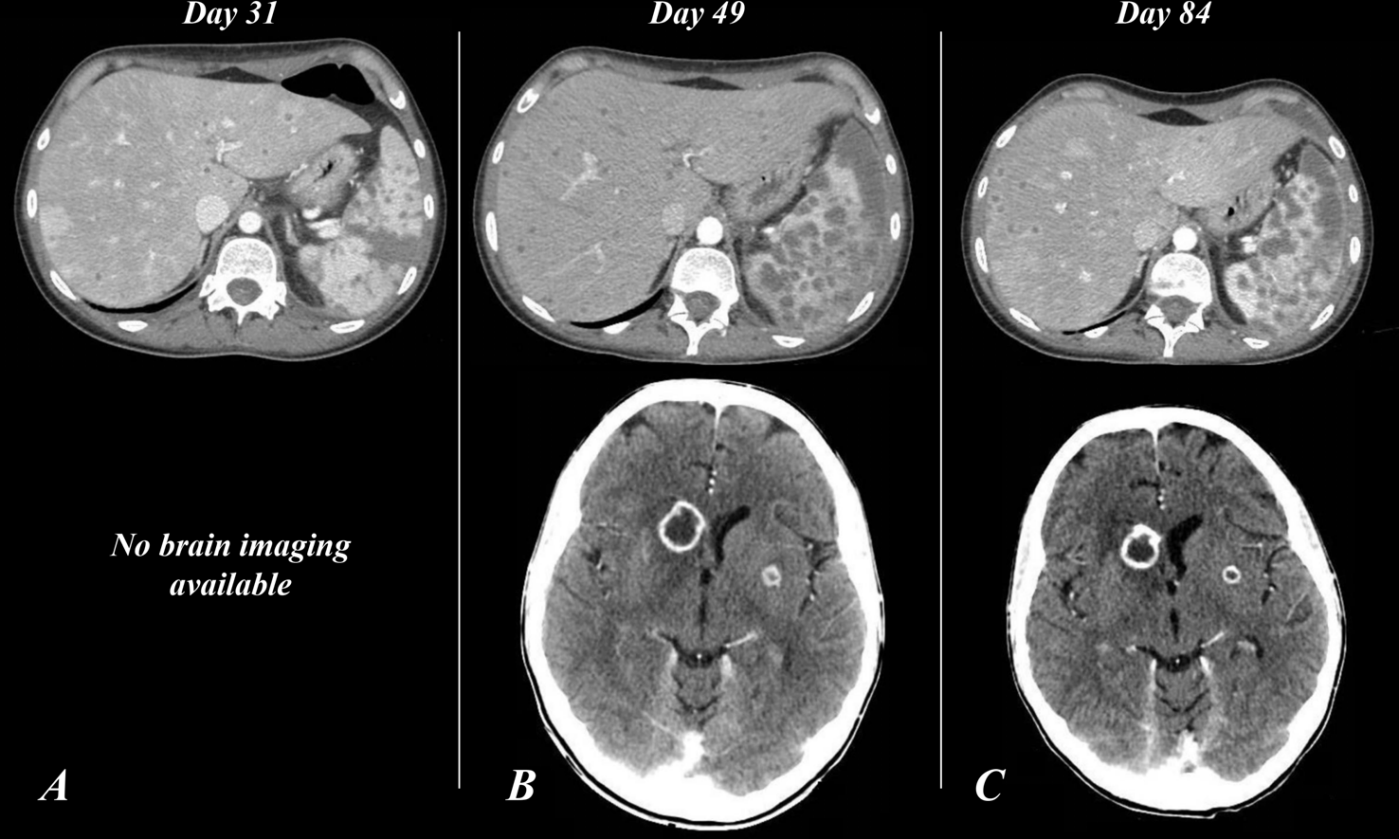
Timeline chart of WBC/ANC and blood cultures, GTX and PCT. Graphic representation of the WBC (blue line), ANC (orange line), and PCT (gray line) curves and their correlation with *Saprochaete clavata* isolation from blood cultures and GTX therapy. 
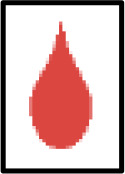
Hemocoltures positive for *Saprochaete clavata*; 
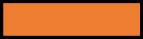
GTX interval. WBC, white blood count; GTX, granulocytes transfusion; PCT, procalcitonin.

Time curves of white blood cells (WBC), absolute neutrophil count (ANC), and procalcitonin (PCT) are shown in **Figure 1**.

On day 21, a high-resolution chest CT (HR-chest-CT) scan revealed a slight ground-glass-like area in the right upper lobe of unspecific significance. Due to persistent fever with negative blood cultures, vancomycin (500 mg/6 h) and liposomal amphotericin B (3 mg/kg/day) were empirically added. On day 28, she was placed on total parenteral nutrition due to grade III (17) mucositis with poor oral intake.

On day 29, *Saprochaete clavata* (*Magnusiomyces clavatus*) was isolated from a blood culture collected from CVC; serum galactomannan antigen (GM) showed a negative result. The susceptibility test showed minimum inhibitory concentration (MIC) values of 1 mg/l for amphotericin B, 1 mg/l for anidulafungin, 8 mg/l for caspofungin, 8 mg/l for fluconazole, 0.06 mg/l for itraconazole, 0.125 mg/l for posaconazole, and 0.125 mg/l for voriconazole. A new HR-chest CT disclosed a thickening of the previous right-upper-lobe finding to a nodule with ground-glass peripheral opacity, consistent with a diagnosis of fungal pneumonia. At this stage, we decided to remove CVC and to potentiate antifungal therapy by escalating the liposomal amphotericin B dosage (from 3 to 5 mg/kg/day) and with the addition of isavuconazole (loading dose of 200 mg/8 h for 48 h, then 200 mg/day). No recent consumption of food suspected of contamination was reported by the patient (10).

**Figure 2:**
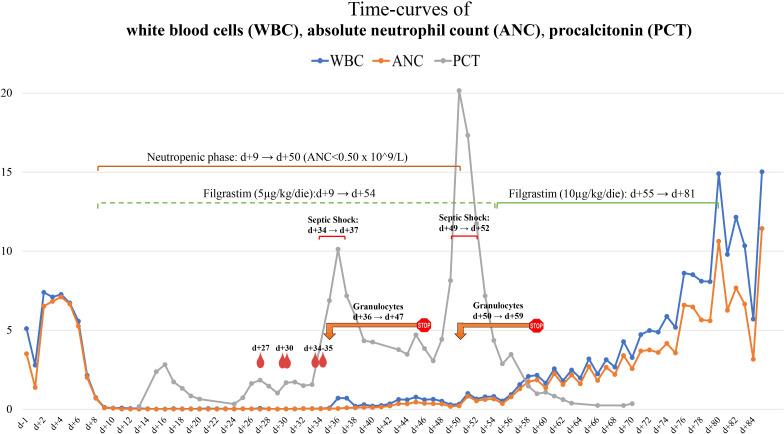
Contrast-enhanced CT scan of the abdomen **(A–C)** and the brain **(B, C)**. Panel A (*day 31*), finding of multiple, sub-centimetric, hypodense lesions involving the spleen and liver parenchymas. **(B)** (*Day 49*), spleen and liver enlargement; increase in number and size of the hypodense round lesions, some with confluent and liquefied patterns; new finding of nodular ring-enhancing lesions of the subcortical CNS with compression of the frontal (anterior) horn of the right lateral ventricle, and involvement of the left-side putamen. **(C)** (*Day 84*), confirmation of abdominal picture; stable brain lesions, expansion of the lesion surrounding with complete flattening of the frontal pole of the right lateral ventricle. CT, computed-tomography; CNS, central nervous system.

On day 31, because of emerging abdominal pain, an abdominal contrast-enhanced CT scan was performed, which documented multiple, sub-centimetric, hypodense, ubiquitously distributed round areas in the spleen and liver (**Figure 2A**) suggesting a disseminated *Saprochaete clavata* infection. A new BMA showed a markedly hypocellular BM, consistent with a persistent post-CHT aplasia.

On day 33, two additional blood cultures (collected from peripheral blood) showed a positive result for *S. clavata*. The day after, the clinical conditions worsened, developing septic shock. Considering the patient’s critical status, the persistence of fever unresponsive to antifungal therapy, and blood culture positivity for *S. clavata*, we decided to screen potential donors for granulocyte harvest and transfusion (13, 16, 18, 19).

On day 35, *S. clavata* was also isolated from stool sample culture. Correlations between significant clinical events and laboratory tests are depicted in **Figure 1**.

On day 36, GTX was initiated and a rapid decrease in inflammatory markers (**Figure 1**) was observed, together with improvement of the patient’s clinical condition. Afterward, no further *S. clavata*-positive hemocultures were found. In contrast with the improvement of clinical and laboratory parameters, the temperature curve did not show any improvement, continuing to exhibit at least two to four fever spikes per day.

On day 40, a new BMA was performed, confirming hypoplasia. Daily GTX was continued until day 47 and was stopped when a stable clinical condition was reached.

Forty-eight hours after GTX discontinuation, the patient rapidly worsened, developing generalized edema, septic shock, and respiratory distress. A total body CT scan displayed a further increase in the size of the lung nodule, now also showing a central necrotizing area and a worsening of the previous reported abdominal hypodense round lesions, with progression to confluent and liquefied patterns. Furthermore, three new nodular ring-enhancing lesions appeared in the subcortical CNS in the absence of neurologic signs or symptoms (**Figure 2B**).

Based on the evidence of infection recrudescence, on day 50, we resumed GTX with a pronounced and prompt improvement of both clinical condition and inflammatory markers (**Figure 1**). Weekly cytomegalovirus (CMV) quantitative molecular testing always showed a negative result. On day 54, a new BMA was consistent with CR.

On day 55 (after 46 days of filgrastim 5 µg/kg/day), we decided to double the filgrastim dose in the attempt to accelerate neutrophil count reconstitution. After 2 days of filgrastim 10 µg/kg/day, ANC exceeded 1.0 × 10^9^/l. GTX was continued until day 59. Indeed, after 21 total granulocyte units, we decided to stop granulocyte transfusion based on substantial clinical stability and ANC steadily at >1.0 × 10^9^/l.

The patient’s general condition gradually improved, and the WBC count stabilized to normal values (**Figure 1**), so we decided to interrupt filgrastim administration (after 46 days with 5 µg/kg/day and 27 days with 10 µg/kg/day) and to repeat a contrast-enhanced total body CT scan (day 84), which showed stable brain lesions and cavitation of the lung nodule together with a significant reduction in pleural, pericardial, and free-abdominal effusions. Also, a marked and diffuse modification of spleen parenchyma (**Figure 2C**) was documented.

Intravenous antifungal combination therapy was administered until the day of discharge (day 86), then continued with oral isavuconazole. She is still in CR, under clinic follow-up, and her clinical condition is steadily improving.

## Discussion and conclusion

FLAI is a well-known CHT regimen, frequently used in treating AML relapse. Burnett et al. (20) showed how the use of FAI as a first-line approach could lead to a greater amount of patients achieving an overall remission and CR after the first course of CHT, with reduction in relapse risk and prolongation of relapse-free survival when compared to standard therapy (20, 21). However, two courses of FLAI are more likely to be associated with greater hematological toxicity, so it should be considered in young patients (20, 21).

In this report, *S. clavata* was found to be responsible for disseminated and life-threatening infection in a young patient with AML, during a profound neutropenic phase after the second course of the FLAI CHT regimen. In line with available literature (10, 12, 22, 23), the susceptibility test has shown a high MIC for echinocandins and fluconazole and low for amphotericin B, itraconazole, posaconazole, and voriconazole (10, 12, 23–25). Not enough clinical data are available to assess the optimal treatment for this infection: a 2014 guideline for treatment of *S. clavata* infection recommended any formulation of amphotericin B ± other antifungals as the preferred approach (12, 22). Isavuconazole MIC was not tested in our case, but considering that the data about susceptibility to this agent are controversial (10, 12, 25), we tried to take advantage of its synergistic action with liposomal amphotericin B similar to other severe fungal infections (26).

The fungus continued to be isolated from blood cultures, and the infection progressed despite the antifungal combination therapy until the administration of GTX. Since the beginning of GTX, *S. clavata* was never isolated again (**Figure 1**). In addition to positive blood cultures, the isolation of *S. clavata* in stool (day 35) could indicate to consider the colonization of the GI tract as a reservoir from which the fungus may have reached the bloodstream (probably during GI mucositis). Serum GM turned out to be an unreliable marker of *S. clavata* infection (12).

Considering the critical conditions of the patient at the time of GTX arrangement, we decided to start screening for donors from both community and related individuals, to have the widest resources to continue GTX until the hematological recovery or complete resolution of the infection. A single granulocyte bag was obtained from each donor (granulocytapheresis), for a total of 21 granulocytes units received during the admission.

The effectiveness of this approach was demonstrated by the clear improvement (**Figure 1**) observed after the first course of GTX and confirmed by the sudden worsening of clinical condition upon GTX suspension. Hematological recovery was essential in restoring clinical condition and normalizing inflammatory markers. This could be the clue that antifungal alone might have a high failure rate unless supported by correcting factors (principally neutropenia) that expose the patients to the progression of the infection (13).

Three BMAs were performed during the hospitalization. The first evaluation (day 30) showed an aplastic BM in the absence of blasts, which was interpreted as a positive finding (AML was not the underlying cause of the delayed hematological recovery) even though a profound myelosuppression after the second course of FLAI was confirmed (20, 21). The two subsequent BMAs were still hypocellular, but both showed signs of initial multilineage recovery of hematopoiesis, without evidence of blast infiltrate. Despite these findings, the ANC curve exceeded 0.5 × 10^9^/l only after 14 granulocyte units, reaching values steadily above 1.0 × 10^9^/l after doubling of the filgrastim dose.

In this report, GTX proved to be a potential additional therapy in treating serious and antifungal-refractory fungal infections during the profound post-CHT neutropenic phase. Therefore, it suggests that this approach could be effective and should be considered in life-threatening infections.

In addition, this case also demonstrates that even combination antifungal therapy might not be sufficient in containing disseminated IFI unless it is accompanied by the patient’s immune system recovery.

## Patient perspective

The patient experienced a hard ordeal which led to a general worsening of her clinical condition (ECOG 2-3), together with a negative psychologic consequence in terms of motivation. Her clinical conditions have now substantially improved. At present, the patient is planned to receive a fully matched unrelated donor hematopoietic stem cell transplantation: her behavior is a mixture of concern and expectation regarding her therapeutic plan.

## Data availability statement

The original contributions presented in the study are included in the article/supplementary material. Further inquiries can be directed to the corresponding author.

## Ethics statement

Written informed consent was obtained from the individual(s) for the publication of any potentially identifiable images or data included in this article.

## Author contributions

All authors contributed to the clinical management of the patient. All authors wrote the manuscript, revised it critically and gave final approval to submit for publication.

## Funding

GS is supported by the Italian Association for Cancer Research (AIRC) (grant IG-2020-24440), the Ministry of the University and Research (MUR)-European Union (TITAN project), and the Rome Foundation.

## Conflict of interest

The authors declare that the research was conducted in the absence of any commercial or financial relationships that may represent a potential conflict of interest.

## Publisher’s note

All claims expressed in this article are solely those of the authors and do not necessarily represent those of their affiliated organizations, or those of the publisher, the editors and the reviewers. Any product that may be evaluated in this article, or claim that may be made by its manufacturer, is not guaranteed or endorsed by the publisher.
